# Potassium *N*-bromo-2-nitro­benzene­sulfonamidate monohydrate

**DOI:** 10.1107/S1600536812042080

**Published:** 2012-10-13

**Authors:** B. Thimme Gowda, Sabine Foro, H. S. Spandana

**Affiliations:** a Department of Chemistry, Mangalore University, Mangalagangotri 574 199, Mangalore, India; b Institute of Materials Science, Darmstadt University of Technology, Petersenstrasse 23, D-64287 Darmstadt, Germany

## Abstract

In the title compound, K^+^·C_6_H_4_BrN_2_O_4_S^−^·H_2_O, the K^+^ ion is hepta-coordinated by two O atoms from two different water mol­ecules, three sulfonyl O atoms from three *N*-bromo-2-nitro-benzene­sulfonamidate anions and two nitro O atoms from two *N*-bromo-2-nitro-benzene­sulfonamidate anions. The S—N distance of 1.576 (4) Å is consistent with an S=N double bond. The crystal structure is stabilized by inter­molecular O—H⋯N and O—H⋯Br hydrogen bonds which link the molecules into polymeric layers running parallel to the *bc* plane.

## Related literature

For the preparation of metal salts of *N*-haloaryl­sulfonamides, see: Gowda & Mahadevappa (1983 ▶); Usha & Gowda (2006 ▶). For studies on the effect of substituents and metal ions on the structures of *N*-haloaryl­sulfonamides, see: George *et al.* (2000 ▶); Gowda *et al.* (2011*a* ▶,*b* ▶); Olmstead & Power (1986 ▶). For positioning of water H atoms, see: Nardelli (1999 ▶).
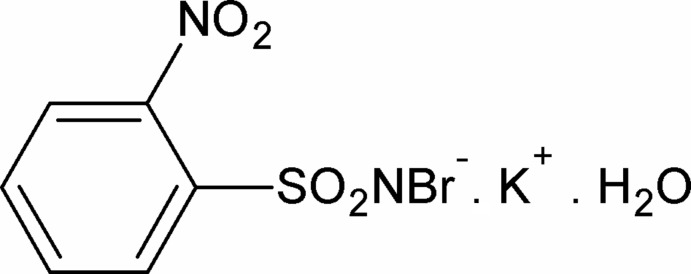


## Experimental

### 

#### Crystal data

K^+^·C_6_H_4_BrN_2_O_4_S^−^·H_2_O
*M* *_r_* = 337.20
Monoclinic, 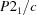

*a* = 13.034 (2) Å
*b* = 12.815 (2) Å
*c* = 6.7741 (9) Å
β = 100.65 (1)°
*V* = 1112.0 (3) Å^3^
*Z* = 4
Mo *K*α radiation
μ = 4.27 mm^−1^
*T* = 293 K
0.48 × 0.48 × 0.24 mm

#### Data collection

Oxford Diffraction Xcalibur diffractometer with Sapphire CCD detector
Absorption correction: multi-scan (*CrysAlis RED*; Oxford Diffraction, 2009 ▶) *T*_min_ = 0.234, *T*_max_ = 0.428
3896 measured reflections
2236 independent reflections
1847 reflections with *I* > 2σ(*I*)
*R*_int_ = 0.042

#### Refinement

*R*[*F* ^2^ > 2σ(*F* ^2^)] = 0.053
*wR*(*F* ^2^) = 0.145
*S* = 1.06
2236 reflections
152 parameters
3 restraints
H atoms treated by a mixture of independent and constrained refinement
Δρ_max_ = 0.79 e Å^−3^
Δρ_min_ = −1.21 e Å^−3^

### 

Data collection: *CrysAlis CCD* (Oxford Diffraction, 2009 ▶); cell refinement: *CrysAlis CCD*; data reduction: *CrysAlis RED* (Oxford Diffraction, 2009 ▶); program(s) used to solve structure: *SHELXS97* (Sheldrick, 2008 ▶); program(s) used to refine structure: *SHELXL97* (Sheldrick, 2008 ▶); molecular graphics: *PLATON* (Spek, 2009 ▶); software used to prepare material for publication: *SHELXL97*.

## Supplementary Material

Crystal structure: contains datablock(s) I, global. DOI: 10.1107/S1600536812042080/zl2509sup1.cif

Structure factors: contains datablock(s) I. DOI: 10.1107/S1600536812042080/zl2509Isup2.hkl

Supplementary material file. DOI: 10.1107/S1600536812042080/zl2509Isup3.cml

Additional supplementary materials:  crystallographic information; 3D view; checkCIF report

## Figures and Tables

**Table 1:** Hydrogen-bond geometry (Å, °)

*D*—H⋯*A*	*D*—H	H⋯*A*	*D*⋯*A*	*D*—H⋯*A*
O5—H51⋯N1^i^	0.84 (2)	2.13 (3)	2.926 (5)	157 (5)
O5—H52⋯Br1^ii^	0.84 (2)	2.85 (4)	3.509 (4)	137 (4)

Symmetry codes: (i) 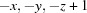; (ii) 
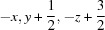
.

**Fig. 1.**
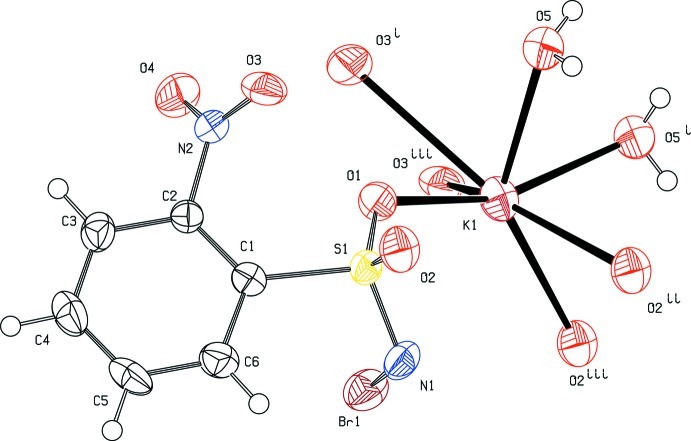
Molecular structure of the title compound, showing the atom labelling scheme for the asymmetric unit and extended to show the coordination geometry for the K+ ion. The displacement ellipsoids are drawn at the 50% probability level. The H atoms are represented as small spheres of arbitrary radii.

**Fig. 2.**
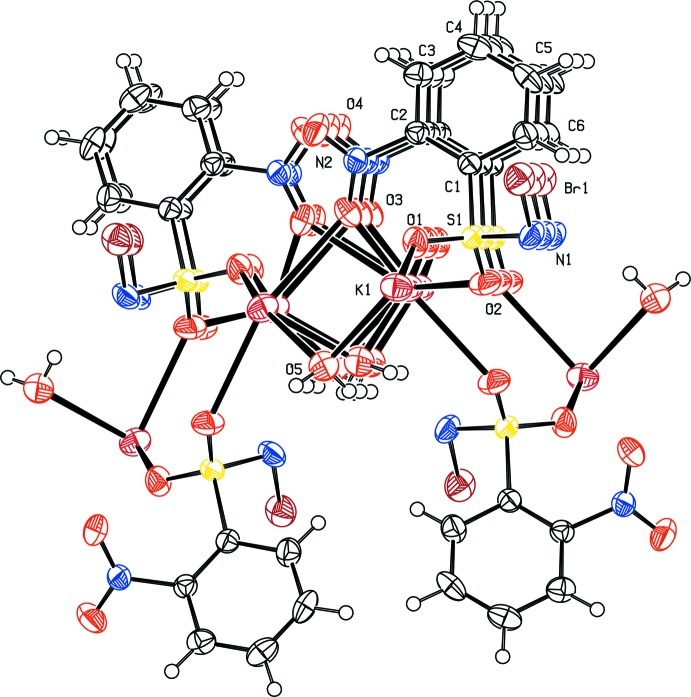
Bridging of potassium cations, N-bromo-2-nitro-benzenesulfonamidate anions and water molecules in the structure of the title compound.

**Fig. 3.**
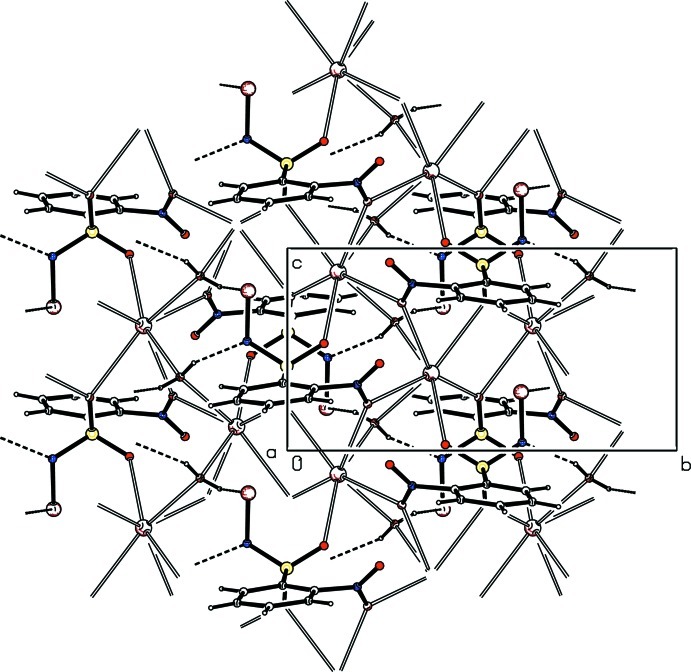
Molecular packing of the title compound with hydrogen bonding shown as dashed lines.

## supplementary crystallographic information

### Comment

In the present work, to explore the effect of substituents on the crystal structures of metal salts of *N*-haloarylsulfonamidates (George *et al.*, 2000; Gowda *et al.*, 2011**a*,b*; Olmstead & Power, 1986), the crystal structure of potassium *N*-bromo-2-nitro-benzenesulfonamidate monohydrate (I) has been determined (Fig. 1). The structure of (I) resembles those of potassium *N*-bromo-2-chloro-benzenesulfonamidate sesquihydrate (II) (Gowda *et al.*, 2011*a*), potassium *N*-bromo-2-methyl-benzenesulfonamidate sesquihydrate (III) (Gowda *et al.*, 2011*b*), and sodium *N*-chloro-arylsulfonamidates (George *et al.*, 2000; Olmstead & Power, 1986).

In the title compound (I), the K^+^ ion is hepta coordinated by two O atoms from two different water molecules, three sulfonyl O atoms from three *N*-bromo-2-nitro-benzenesulfonamidate anions and two nitro O atoms from two *N*-bromo-2-nitro-benzenesulfonamidate anions (Fig 2.). This is in contrast to K^+^ ion hepta coordination by three O atoms from water molecules and by four sulfonyl O atoms of three *N*-bromo-2-chloro-benzenesulfonamide anions in (II) and three *N*-bromo-2-methyl-benzenesulfonamide anions in (III).

The S—N distance of 1.576 (4) Å in (I) is consistent with an S—N double bond and is in agreement with the observed values of 1.582 (4) Å in (II) and 1.577 (5) Å in (III).

The packing diagram consists of a two-dimensional polymeric layer running parallel to the *bc* plane (Fig. 2). The molecular packing is stabilized by O5—H51···N1 and O5—H52···Br1 hydrogen bonds (Table 1).

### Experimental

The title compound was prepared by a method similar to the one described by Gowda & Mahadevappa (Gowda & Mahadevappa, 1983) and Usha & Gowda (Usha & Gowda, 2006). 2 g of 2-nitrobenzenesulfonamide was dissolved with stirring in 40 ml of 5*M* KOH at room temperature. The resultant solution was cooled in ice and 4 ml of liquid bromine was added drop wise with constant stirring. The resultant potassium salt of *N*-bromo-2-nitro-benzenesulfonamidate monohydrate was filtered under suction, washed quickly with a minimum quantity of ice cold water. The purity of the compound was checked by determining its melting point (175–177°C) and estimating, iodometrically, the amount of active bromine present in it. It was further characterized from its infrared spectrum. The characteristic absorptions observed are 3624.3, 3333.0, 3192.2, 2978.1, 2922.2, 2075.4, 1626.0, 1602.9, 1477.5, 1452.4, 1242.2, 1122.6, 1060.9, 937.4, 817.8, 686.7, 640.4, 578.6, 549.6, 524.6 and 470.6 cm^-1^.

Prism like yellow single crystals of the title compound used in the X-ray diffraction studies were obtained from its aqueous solution at room temperature.

### Refinement

H atoms bonded to C were positioned with idealized geometry using a riding model with the aromatic C—H = 0.93 Å. The O-bound H atoms were located in a difference map and were refined with restrained geometry (Nardelli, 1999), *viz*. O—H distances were restrained to 0.85 (2) Å and the H—H distance was restrained to 1.365 Å, thus leading to the angle of 107°. All H atoms were refined with isotropic displacement parameters set at 1.2 *U*_eq_ of the parent atom. The residual electron-density features are located in the region of Br1. The highest peak and the deepest hole are 0.80 and 0.84 Å from Br1, respectivily.

### Crystal data

**Table:**

K^+^·C_6_H_4_BrN_2_O_4_S^−^·H_2_O	*F*(000) = 664
*M**_r_* = 337.20	*D*_x_ = 2.014 Mg m^−^^3^
Monoclinic, *P*2_1_/*c*	Mo *K*α radiation, λ = 0.71073 Å
Hall symbol: -P 2ybc	Cell parameters from 2399 reflections
*a* = 13.034 (2) Å	θ = 3.1–27.8°
*b* = 12.815 (2) Å	µ = 4.27 mm^−^^1^
*c* = 6.7741 (9) Å	*T* = 293 K
β = 100.65 (1)°	Prism, yellow
*V* = 1112.0 (3) Å^3^	0.48 × 0.48 × 0.24 mm
*Z* = 4	

### Data collection

**Table:**

Oxford Diffraction Xcalibur diffractometer with Sapphire CCD detector	2236 independent reflections
Radiation source: fine-focus sealed tube	1847 reflections with *I* > 2σ(*I*)
Graphite monochromator	*R*_int_ = 0.042
Rotation method data acquisition using ω scans.	θ_max_ = 26.4°, θ_min_ = 3.2°
Absorption correction: multi-scan (*CrysAlis RED*; Oxford Diffraction, 2009)	*h* = −16→12
*T*_min_ = 0.234, *T*_max_ = 0.428	*k* = −16→11
3896 measured reflections	*l* = −6→8

### Refinement

**Table:**

Refinement on *F*^2^	Secondary atom site location: difference Fourier map
Least-squares matrix: full	Hydrogen site location: inferred from neighbouring sites
*R*[*F*^2^ > 2σ(*F*^2^)] = 0.053	H atoms treated by a mixture of independent and constrained refinement
*wR*(*F*^2^) = 0.145	*w* = 1/[σ^2^(*F*_o_^2^) + (0.0909*P*)^2^ + 0.1082*P*] where *P* = (*F*_o_^2^ + 2*F*_c_^2^)/3
*S* = 1.06	(Δ/σ)_max_ = 0.002
2236 reflections	Δρ_max_ = 0.79 e Å^−^^3^
152 parameters	Δρ_min_ = −1.21 e Å^−^^3^
3 restraints	Extinction correction: *SHELXL97* (Sheldrick, 2008), Fc^*^=kFc[1+0.001xFc^2^λ^3^/sin(2θ)]^-1/4^
Primary atom site location: structure-invariant direct methods	Extinction coefficient: 0.082 (5)

### Special details

**Table:**

Geometry. All e.s.d.'s (except the e.s.d. in the dihedral angle between two l.s. planes) are estimated using the full covariance matrix. The cell e.s.d.'s are taken into account individually in the estimation of e.s.d.'s in distances, angles and torsion angles; correlations between e.s.d.'s in cell parameters are only used when they are defined by crystal symmetry. An approximate (isotropic) treatment of cell e.s.d.'s is used for estimating e.s.d.'s involving l.s. planes.
Refinement. Refinement of *F*^2^ against ALL reflections. The weighted *R*-factor *wR* and goodness of fit *S* are based on *F*^2^, conventional *R*-factors *R* are based on *F*, with *F* set to zero for negative *F*^2^. The threshold expression of *F*^2^ > σ(*F*^2^) is used only for calculating *R*-factors(gt) *etc*. and is not relevant to the choice of reflections for refinement. *R*-factors based on *F*^2^ are statistically about twice as large as those based on *F*, and *R*- factors based on ALL data will be even larger.

### Fractional atomic coordinates and isotropic or equivalent isotropic displacement parameters (Å^2^)

**Table:**

	*x*	*y*	*z*	*U*_iso_*/*U*_eq_	
C1	0.2873 (3)	−0.0123 (3)	0.3340 (6)	0.0272 (9)	
C2	0.3529 (3)	0.0693 (3)	0.3079 (6)	0.0271 (8)	
C3	0.4505 (3)	0.0546 (4)	0.2573 (6)	0.0346 (10)	
H3	0.4930	0.1113	0.2430	0.042*	
C4	0.4833 (4)	−0.0463 (4)	0.2286 (7)	0.0419 (11)	
H4	0.5488	−0.0583	0.1975	0.050*	
C5	0.4173 (4)	−0.1291 (4)	0.2469 (7)	0.0425 (11)	
H5	0.4377	−0.1966	0.2221	0.051*	
C6	0.3214 (4)	−0.1127 (3)	0.3015 (7)	0.0368 (10)	
H6	0.2791	−0.1695	0.3167	0.044*	
Br1	0.24804 (4)	−0.10055 (4)	0.78959 (7)	0.0470 (3)	
K1	0.09562 (9)	0.13045 (8)	0.88211 (15)	0.0418 (3)	
N1	0.1510 (3)	−0.1035 (3)	0.5411 (6)	0.0384 (9)	
N2	0.3214 (3)	0.1792 (3)	0.3257 (5)	0.0316 (8)	
O1	0.1685 (3)	0.0941 (2)	0.5295 (5)	0.0395 (8)	
O2	0.0840 (3)	−0.0085 (2)	0.2371 (5)	0.0442 (8)	
O3	0.2363 (3)	0.2061 (3)	0.2308 (5)	0.0454 (8)	
O4	0.3826 (3)	0.2369 (3)	0.4304 (6)	0.0520 (9)	
S1	0.16307 (8)	−0.00155 (7)	0.41728 (15)	0.0294 (3)	
O5	−0.0180 (3)	0.2758 (3)	0.6363 (6)	0.0494 (9)	
H51	−0.066 (3)	0.240 (3)	0.566 (7)	0.059*	
H52	−0.044 (4)	0.326 (3)	0.689 (8)	0.059*	

### Atomic displacement parameters (Å^2^)

**Table:**

	*U*^11^	*U*^22^	*U*^33^	*U*^12^	*U*^13^	*U*^23^
C1	0.027 (2)	0.0276 (19)	0.0253 (18)	0.0017 (17)	0.0016 (16)	−0.0018 (16)
C2	0.026 (2)	0.0279 (18)	0.0271 (18)	0.0015 (17)	0.0053 (16)	0.0028 (16)
C3	0.032 (2)	0.042 (2)	0.032 (2)	0.000 (2)	0.0101 (18)	0.0059 (19)
C4	0.036 (3)	0.054 (3)	0.038 (2)	0.014 (2)	0.015 (2)	0.001 (2)
C5	0.047 (3)	0.036 (2)	0.045 (3)	0.020 (2)	0.012 (2)	−0.003 (2)
C6	0.041 (3)	0.030 (2)	0.040 (2)	0.0028 (19)	0.009 (2)	−0.0020 (18)
Br1	0.0516 (4)	0.0461 (4)	0.0432 (4)	−0.0034 (2)	0.0087 (2)	0.0096 (2)
K1	0.0378 (6)	0.0498 (6)	0.0380 (5)	0.0041 (5)	0.0076 (4)	−0.0024 (5)
N1	0.036 (2)	0.034 (2)	0.047 (2)	−0.0106 (16)	0.0122 (19)	0.0010 (16)
N2	0.032 (2)	0.0279 (17)	0.0369 (18)	−0.0005 (15)	0.0100 (16)	0.0052 (15)
O1	0.046 (2)	0.0313 (15)	0.0463 (18)	−0.0007 (13)	0.0206 (16)	−0.0100 (13)
O2	0.0293 (17)	0.0527 (19)	0.0465 (18)	−0.0035 (15)	−0.0039 (15)	−0.0014 (16)
O3	0.045 (2)	0.0325 (17)	0.0564 (19)	0.0096 (15)	0.0025 (16)	0.0113 (15)
O4	0.048 (2)	0.0379 (17)	0.070 (2)	−0.0146 (16)	0.0112 (19)	−0.0130 (17)
S1	0.0254 (6)	0.0274 (5)	0.0360 (5)	−0.0033 (4)	0.0069 (4)	−0.0032 (4)
O5	0.043 (2)	0.0443 (19)	0.063 (2)	−0.0011 (16)	0.0146 (18)	0.0010 (17)

### Geometric parameters (Å, º)

**Table:**

C1—C2	1.383 (6)	K1—O2^ii^	2.806 (3)
C1—C6	1.391 (5)	K1—O3^iii^	2.877 (4)
C1—S1	1.815 (4)	K1—O2^iii^	3.018 (4)
C2—C3	1.390 (6)	K1—O3^i^	3.081 (4)
C2—N2	1.479 (5)	K1—H51	3.06 (5)
C3—C4	1.386 (6)	N1—S1	1.576 (4)
C3—H3	0.9300	N2—O4	1.215 (5)
C4—C5	1.385 (7)	N2—O3	1.225 (5)
C4—H4	0.9300	O1—S1	1.437 (3)
C5—C6	1.384 (7)	O2—S1	1.448 (3)
C5—H5	0.9300	O2—K1^ii^	2.806 (3)
C6—H6	0.9300	O2—K1^iv^	3.018 (4)
Br1—N1	1.910 (4)	O3—K1^iv^	2.877 (4)
Br1—K1	3.6829 (12)	O3—K1^v^	3.081 (4)
K1—O5	2.743 (4)	O5—K1^v^	2.746 (4)
K1—O5^i^	2.746 (4)	O5—H51	0.844 (19)
K1—O1	2.768 (3)	O5—H52	0.841 (19)
			
C2—C1—C6	117.1 (4)	O2^iii^—K1—O3^i^	141.88 (10)
C2—C1—S1	126.2 (3)	O5—K1—Br1	133.65 (9)
C6—C1—S1	116.7 (3)	O5^i^—K1—Br1	145.95 (9)
C1—C2—C3	123.0 (4)	O1—K1—Br1	55.66 (7)
C1—C2—N2	121.5 (4)	O2^ii^—K1—Br1	87.19 (8)
C3—C2—N2	115.4 (4)	O3^iii^—K1—Br1	97.38 (7)
C4—C3—C2	118.7 (4)	O2^iii^—K1—Br1	76.67 (7)
C4—C3—H3	120.6	O3^i^—K1—Br1	96.72 (7)
C2—C3—H3	120.6	O5—K1—H51	15.6 (6)
C5—C4—C3	119.3 (4)	O5^i^—K1—H51	81.7 (9)
C5—C4—H4	120.4	O1—K1—H51	77.0 (10)
C3—C4—H4	120.4	O2^ii^—K1—H51	68.0 (7)
C6—C5—C4	120.9 (4)	O3^iii^—K1—H51	132.2 (6)
C6—C5—H5	119.5	O2^iii^—K1—H51	134.5 (10)
C4—C5—H5	119.5	O3^i^—K1—H51	80.1 (8)
C5—C6—C1	120.9 (4)	Br1—K1—H51	125.1 (8)
C5—C6—H6	119.5	S1—N1—Br1	109.8 (2)
C1—C6—H6	119.5	O4—N2—O3	124.7 (4)
N1—Br1—K1	82.87 (12)	O4—N2—C2	117.7 (4)
O5—K1—O5^i^	77.92 (8)	O3—N2—C2	117.6 (3)
O5—K1—O1	79.82 (11)	S1—O1—K1	127.53 (18)
O5^i^—K1—O1	157.72 (11)	S1—O2—K1^ii^	134.68 (19)
O5—K1—O2^ii^	82.86 (11)	S1—O2—K1^iv^	120.16 (18)
O5^i^—K1—O2^ii^	84.62 (11)	K1^ii^—O2—K1^iv^	105.16 (10)
O1—K1—O2^ii^	93.37 (11)	N2—O3—K1^iv^	135.6 (3)
O5—K1—O3^iii^	117.39 (11)	N2—O3—K1^v^	123.6 (3)
O5^i^—K1—O3^iii^	70.94 (11)	K1^iv^—O3—K1^v^	100.02 (11)
O1—K1—O3^iii^	119.80 (11)	O1—S1—O2	117.1 (2)
O2^ii^—K1—O3^iii^	142.64 (11)	O1—S1—N1	115.2 (2)
O5—K1—O2^iii^	141.57 (11)	O2—S1—N1	105.8 (2)
O5^i^—K1—O2^iii^	69.28 (11)	O1—S1—C1	105.67 (19)
O1—K1—O2^iii^	131.59 (10)	O2—S1—C1	105.7 (2)
O2^ii^—K1—O2^iii^	74.84 (10)	N1—S1—C1	106.5 (2)
O3^iii^—K1—O2^iii^	70.30 (9)	K1—O5—K1^v^	112.63 (13)
O5—K1—O3^i^	67.90 (10)	K1—O5—H51	104 (4)
O5^i^—K1—O3^i^	109.52 (11)	K1^v^—O5—H51	108 (4)
O1—K1—O3^i^	60.50 (9)	K1—O5—H52	118 (4)
O2^ii^—K1—O3^i^	143.02 (10)	K1^v^—O5—H52	104 (4)
O3^iii^—K1—O3^i^	73.50 (8)	H51—O5—H52	110 (3)
			
C6—C1—C2—C3	−1.9 (6)	O4—N2—O3—K1^iv^	156.5 (3)
S1—C1—C2—C3	175.3 (3)	C2—N2—O3—K1^iv^	−22.1 (5)
C6—C1—C2—N2	176.0 (4)	O4—N2—O3—K1^v^	−35.7 (5)
S1—C1—C2—N2	−6.8 (6)	C2—N2—O3—K1^v^	145.7 (3)
C1—C2—C3—C4	1.0 (6)	K1—O1—S1—O2	102.7 (3)
N2—C2—C3—C4	−177.0 (4)	K1—O1—S1—N1	−22.6 (3)
C2—C3—C4—C5	1.3 (7)	K1—O1—S1—C1	−139.9 (2)
C3—C4—C5—C6	−2.8 (7)	K1^ii^—O2—S1—O1	−114.6 (3)
C4—C5—C6—C1	1.8 (8)	K1^iv^—O2—S1—O1	65.8 (3)
C2—C1—C6—C5	0.5 (7)	K1^ii^—O2—S1—N1	15.4 (3)
S1—C1—C6—C5	−177.0 (4)	K1^iv^—O2—S1—N1	−164.3 (2)
N1—Br1—K1—O5	−28.49 (17)	K1^ii^—O2—S1—C1	128.1 (3)
N1—Br1—K1—O5^i^	124.97 (19)	K1^iv^—O2—S1—C1	−51.6 (2)
N1—Br1—K1—O1	−47.15 (14)	Br1—N1—S1—O1	−46.5 (3)
N1—Br1—K1—O2^ii^	48.87 (14)	Br1—N1—S1—O2	−177.5 (2)
N1—Br1—K1—O3^iii^	−168.38 (14)	Br1—N1—S1—C1	70.3 (2)
N1—Br1—K1—O2^iii^	123.95 (13)	C2—C1—S1—O1	−24.4 (4)
N1—Br1—K1—O3^i^	−94.22 (13)	C6—C1—S1—O1	152.8 (3)
K1—Br1—N1—S1	63.0 (2)	C2—C1—S1—O2	100.4 (4)
C1—C2—N2—O4	131.2 (4)	C6—C1—S1—O2	−82.4 (4)
C3—C2—N2—O4	−50.8 (5)	C2—C1—S1—N1	−147.4 (4)
C1—C2—N2—O3	−50.2 (5)	C6—C1—S1—N1	29.8 (4)
C3—C2—N2—O3	127.9 (4)	O5^i^—K1—O5—K1^v^	140.66 (16)
O5—K1—O1—S1	−120.6 (3)	O1—K1—O5—K1^v^	−38.60 (13)
O5^i^—K1—O1—S1	−122.5 (3)	O2^ii^—K1—O5—K1^v^	−133.34 (15)
O2^ii^—K1—O1—S1	−38.5 (3)	O3^iii^—K1—O5—K1^v^	79.80 (16)
O3^iii^—K1—O1—S1	123.5 (2)	O2^iii^—K1—O5—K1^v^	172.23 (12)
O2^iii^—K1—O1—S1	34.2 (3)	O3^i^—K1—O5—K1^v^	23.55 (11)
O3^i^—K1—O1—S1	169.1 (3)	Br1—K1—O5—K1^v^	−54.17 (18)
Br1—K1—O1—S1	45.8 (2)		

Symmetry codes: (i) *x*, −*y*+1/2, *z*+1/2; (ii) −*x*, −*y*, −*z*+1; (iii) *x*, *y*, *z*+1; (iv) *x*, *y*, *z*−1; (v) *x*, −*y*+1/2, *z*−1/2.

### Hydrogen-bond geometry (Å, º)

**Table:**

*D*—H···*A*	*D*—H	H···*A*	*D*···*A*	*D*—H···*A*
O5—H51···N1^ii^	0.84 (2)	2.13 (3)	2.926 (5)	157 (5)
O5—H52···Br1^vi^	0.84 (2)	2.85 (4)	3.509 (4)	137 (4)

Symmetry codes: (ii) −*x*, −*y*, −*z*+1; (vi) −*x*, *y*+1/2, −*z*+3/2.

## References

[bb1] George, E., Vivekanandan, S. & Sivakumar, K. (2000). *Acta Cryst.* C**56**, 1208–1209. 10.1107/s0108270100007617 11025298

[bb2] Gowda, B. T., Foro, S. & Shakuntala, K. (2011*a*). *Acta Cryst.* E**67**, m926. 10.1107/S1600536811022136 PMC3151922 21836910

[bb3] Gowda, B. T., Foro, S. & Shakuntala, K. (2011*b*). *Acta Cryst.* E**67**, m1015. 10.1107/S1600536811025153 PMC3212111 22090813

[bb4] Gowda, B. T. & Mahadevappa, D. S. (1983). *Talanta*, **30**, 359–362. 10.1016/0039-9140(83)80080-0 18963373

[bb5] Nardelli, M. (1999). *J. Appl. Cryst.* **32**, 563–571.

[bb6] Olmstead, M. M. & Power, P. P. (1986). *Inorg. Chem.* **25**, 4057–4058.

[bb7] Oxford Diffraction (2009). *CrysAlis CCD* and *CrysAlis RED* Oxford Diffraction Ltd, Abingdon, England.

[bb8] Sheldrick, G. M. (2008). *Acta Cryst.* A**64**, 112–122. 10.1107/S0108767307043930 18156677

[bb9] Spek, A. L. (2009). *Acta Cryst.* D**65**, 148–155. 10.1107/S090744490804362X PMC2631630 19171970

[bb10] Usha, K. M. & Gowda, B. T. (2006). *J. Chem. Sci.* **118**, 351–359.

